# Reducing cardiometabolic risk in adults with a low socioeconomic position: protocol of the Supreme Nudge parallel cluster-randomised controlled supermarket trial

**DOI:** 10.1186/s12937-020-00562-8

**Published:** 2020-05-19

**Authors:** Josine M. Stuber, Joreintje D. Mackenbach, Femke E. de Boer, Gert-Jan de Bruijn, Marleen Gillebaart, Marjolein C. Harbers, Jody C. Hoenink, Michel C. A. Klein, Cédric N. H. Middel, Yvonne T. van der Schouw, Tjerk Jan Schuitmaker-Warnaar, Elizabeth Velema, Anne L. Vos, Wilma E. Waterlander, Jeroen Lakerveld, Joline W. J. Beulens

**Affiliations:** 1 Department of Epidemiology and Biostatistics, Amsterdam Public Health research institute, Amsterdam UMC, VU University Amsterdam, Amsterdam, the Netherlands; 2 Upstream Team, www.upstreamteam.nl, Amsterdam UMC, VU University Amsterdam, Amsterdam, the Netherlands; 3 Department of Social, Health and Organizational Psychology, Utrecht University, Utrecht, the Netherlands; 4 Amsterdam School of Communication Research ASCoR, University of Amsterdam, Amsterdam, the Netherlands; 5 Julius Center for Health Sciences and Primary Care, University Medical Center Utrecht, Utrecht University, Utrecht, the Netherlands; 6 Social AI group, department of Computer Science, VU University Amsterdam, Amsterdam, the Netherlands; 7 Athena Institute, Faculty of Science, VU University, Amsterdam, The Netherlands; 8 Netherlands Nutrition Centre (Voedingscentrum), The Hague, The Netherlands; 9 Department of Public Health, Amsterdam Public Health research institute, Amsterdam UMC, University of Amsterdam, Amsterdam, the Netherlands

**Keywords:** Cardiovascular disease, Type 2 diabetes, Food environment, mHealth, eHealth, Socioeconomic status

## Abstract

**Background:**

Unhealthy lifestyle behaviours such as unhealthy dietary intake and insufficient physical activity (PA) tend to cluster in adults with a low socioeconomic position (SEP), putting them at high cardiometabolic disease risk. Educational approaches aiming to improve lifestyle behaviours show limited effect in this population. Using environmental and context-specific interventions may create opportunities for sustainable behaviour change. In this study protocol, we describe the design of a real-life supermarket trial combining nudging, pricing and a mobile PA app with the aim to improve lifestyle behaviours and lower cardiometabolic disease risk in adults with a low SEP.

**Methods:**

The Supreme Nudge trial includes nudging and pricing strategies cluster-randomised on the supermarket level, with: i) control group receiving no intervention; ii) group 1 receiving healthy food nudges (e.g., product placement or promotion); iii) group 2 receiving nudges and pricing strategies (taxing of unhealthy foods and subsidizing healthy foods). In collaboration with a Dutch supermarket chain we will select nine stores located in low SEP neighbourhoods, with the nearest competitor store at > 1 km distance and managed by a committed store manager. Across the clusters, a personalized mobile coaching app targeting walking behaviour will be randomised at the individual level, with: i) control group; ii) a group receiving the mobile PA app. All participants (target *n* = 1485) should be Dutch-speaking, aged 45–75 years with a low SEP and purchase more than half of their household grocery shopping at the selected supermarkets. Participants will be recruited via advertisements and mail-invitations followed by community-outreach methods. Primary outcomes are changes in systolic blood pressure, LDL-cholesterol, HbA1c and dietary intake after 12 months follow-up. Secondary outcomes are changes in diastolic blood pressure, blood lipid markers, waist circumference, steps per day, and behavioural factors including healthy food purchasing, food decision style, social cognitive factors related to nudges and to walking behaviours and customer satisfaction after 12 months follow-up. The trial will be reflexively monitored to support current and future implementation.

**Discussion:**

The findings can guide future research and public health policies on reducing lifestyle-related health inequalities, and contribute to a supermarket-based health promotion intervention implementation roadmap.

**Trial registration:**

Dutch Trial Register ID NL7064, 30th of May, 2018

## Background

The growing prevalence of cardiometabolic diseases (CMDs), including cardiovascular diseases and type 2 diabetes, undermines social and economic development worldwide [1]. Major contributors to the development of CMDs include obesity, high blood pressure, hyperlipidaemia and elevated glucose levels [2]. Unhealthy lifestyle behaviours such as unhealthy dietary intake and insufficient physical activity (PA) increase CMD risk factors and tend to cluster in adults with a low socioeconomic position (SEP) [3–5].

Lifestyle interventions targeting healthy dietary choices and PA through counselling or information provision can be effective in reducing CMD risk factors [6–10]. However, adoption and maintenance of a healthier lifestyle is challenging for most people, especially after interventions cease and for those with a lower SEP [11]. Moreover, educational strategies do not always sufficiently reach those with a lower SEP as interpretation of the information provided requires certain levels of commitment, understanding and motivation. These approaches may therefore widen the existing health inequalities between populations with different SEP levels [12]*.* Using environmental and context-specific (e.g., taking geographic location and its behavioural change opportunities into account) interventions may create opportunities for sustainable behaviour change and better reach all individuals within a population – including those with a lower SEP [13, 14].

Supermarkets provide an important setting for environmental interventions to improve healthy dietary behaviour, as a majority of food choices are made in supermarkets. In Europe, supermarkets are responsible for up to 80% of food sales for home preparation [15]. Supermarket interventions focussing on nudging and pricing have shown to be effective in stimulating healthier food purchases [16]. Nudging refers to environmental changes which make the healthy choice the easier choice, without removing the unhealthy options [17]. Pricing strategies such as subsidies contribute to improving the affordability of healthy foods [18].

Nudging and pricing strategies may be especially effective in individuals with a low SEP. Scarcity of time and resources [19] may lead to a short-term view and increased stress levels [20], which in turn may lead to more automatic and impulsive decision-making [21, 22]. As nudges target heuristic choices and instinctive decision-making [23, 24], they are likely to be particularly effective for those individuals experiencing scarcity circumstances. Current literature indicates that healthy food nudges might be more effective among those with a higher deprivation status, a lower educational level, food insecurity and when on a food assistance program [25–28]. However, heterogeneity of the used SEP proxies hinders adequate comparison of study findings. Current literature regarding the effectiveness of pricing strategies shows that subsidies on healthy products [29–31] and taxes on unhealthy products [32, 33] seem to have similar effects among different SEP levels with possible greater benefits for the groups with a lower SEP [34–37]. However, whether nudging and pricing interventions in the supermarket indeed lead to sustainable behaviour change and improvement of cardiometabolic health is currently unknown [16, 26, 38].

The effects of price increases in the supermarket has never been tested in a real-life controlled trial, nor with a primary focus on populations with a low SEP. Previous supermarket studies have mainly focussed on short-term changes in food purchases, have investigated nudging or pricing strategies as single interventions, and have often only targeted one major food group such as fruits and vegetables [16, 38–40]. Nevertheless, most of these previous studies reported small favourable effects on healthy food purchasing [16]. Such small improvements in healthy food consumption could lead to a small individual decrease in CMD risk and a favourable shift in CMD risk at population level [41].

To promote physical activity, there is an increasing focus on using environmental cues to promote physical activity [42], such as point-of-choice prompt signs that stimulate stair use. Such environmental cues provide relevant information in the environment when the choice is considered. These point-of-choice prompts may be viewed as reminder nudges, as they remind individuals of their own intentions at decision-making moments [43]. As a consequence, these nudges require individuals to have a prior intention to change their physical activity levels in order to be effective [44].

Using mobile coaching techniques that provide personalized messages on specific moments of choice (i.e., just-in-time) is a promising approach to increase PA among a low SEP population. When smartphones of participants and existing modifiable app-platforms are used, messages can be tailored to individual preferences and geographical contexts, and implemented at a relatively low cost. In the Netherlands, smartphone possession increased from 47% in 2012 to 80% in 2017 among individuals with a low educational level [45]. A focus on walking behaviours may be especially suitable for those with a lower SEP given the health benefits of walking and the fact that this is free of costs and can be incorporated within a regular daily routine.

Promoting PA via mobile apps may be effective in increasing the daily step count [46, 47]. However, most mobile PA apps are targeted at – and generally more effective among – individuals with higher levels of health consciousness and motivation to be physically active [48–51]. Also, current PA apps do not take into account the PA opportunities users come across throughout the day within their physical environments, such as green space. Context-specific messages through mobile coaching may enhance the context-action relationship and therefore favourable PA habit formation in a population with a low SEP [52]. The long-term effectiveness of such single prompt signs on CMD risk have not yet been determined in high quality studies. Whether effects spill-over to other environmental contexts that do not have these prompts and their relevance with regard to sustainable improvement of overall PA behaviours remains to be investigated [53].

In this study protocol, we describe the design of a 12-month real-life parallel cluster-randomised controlled supermarket trial combining nudging, pricing and a just-in-time mobile PA coaching app with the aim of improving lifestyle behaviours and lower CMD risk in populations with a low SEP. Furthermore, we aim to contribute to an implementation roadmap to guide future supermarket-based health interventions. This trial is embedded within the larger Supreme Nudge project of which the overarching relevance and design has previously been described [54]. The current protocol focuses on participant-level related outcomes. Within the Supreme Nudge trial, we will also explore retail-related outcomes such as changes in store-level sales trends.

## Methods

### Trial design

**Fig. 1:**
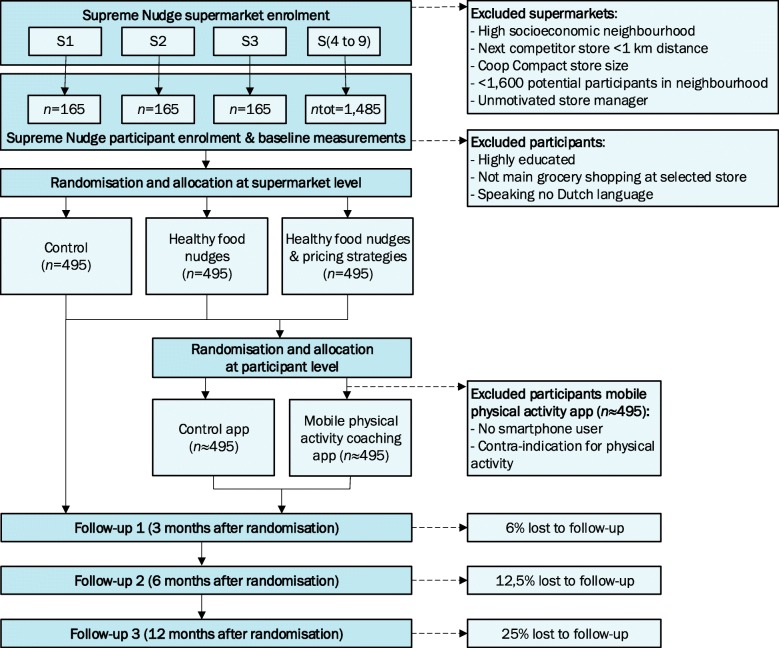
Design of the SUPREME NUDGE cluster-randomised controlled supermarket trial with target recruitment numbers and estimated loss to follow-up

This 12-month cluster-randomised controlled trial (Fig. 1) will implement nudging and pricing strategies on the supermarket level, using three trial arms:

- control group receiving no intervention;
- intervention group 1 receiving healthy food nudges only;
- intervention group 2 receiving healthy food nudges and pricing strategies.

A mobile PA coaching app will be randomised at the individual level across all supermarket clusters to:

- control group receiving a step counter app;
- an intervention group receiving a step counter app plus the mobile PA coaching app.

The trial design deviates from the initial plan as described in the Supreme Nudge project design paper [54], in which the mobile PA app was treated as cluster-randomised intervention arm. However, smartphone ownership could hinder recruitment of participants in the trial and the approach unnecessarily randomised the individual-level PA app intervention at the cluster level, thereby increasing the required sample size. We therefore decided to randomise exposure to the PA app on an individual level.

### Participants

Potential participants have to meet all of the following criteria in order to be eligible for study inclusion:

- have a low SEP (defined as having a practical vocational education level or lower and living in the low SEP neighbourhood surrounding the selected store);
- are aged 45–75 years;
- self-report to do (or report their partner does) more than half of the household grocery shopping at the selected supermarket and plan to continue visiting for the next year;
- provide written informed consent.

Both partners within a single household can be included considering all members of a single household will be exposed to the purchased groceries. By including both partners, we will be able to include a representative population sample allowing for study evaluation that can be translated to possible health benefits on a population level. In order to be eligible for additional inclusion in the PA coaching app intervention, a participant must indicate that they own an Android or iPhone smartphone and use it for text messaging on a regular basis. Potential participants who are not able to adequately communicate in the Dutch language will be excluded from the study. Those who are unable to climb a flight of stairs or have a contra-indication to engage in light PA will be excluded from the mobile PA app intervention.

### Supermarkets

Coop – one of the major supermarket chains in the Netherlands – is a partner in the Supreme Nudge project since the conception of the project idea. Promoting healthy food purchases is one of Coop’s corporate social responsibility goals. This goal is in line with the National Prevention Agreement (2019) by the Dutch Ministry of Health, Welfare and Sport, which describes that supermarkets should entice consumers to buy more healthy products to improve population dietary behaviours. In collaboration with Coop, the supermarket intervention components are co-created and will be implemented in a real-life supermarket setting. Coop currently has over 300 supermarket locations in the Netherlands. Nine supermarkets that meet all of the following criteria will be included in the study:

- located in a low SEP neighbourhood (below average postal code SEP-scores of The Netherlands Institute for Social Research [55]);
- nearest competitor supermarket is at > 1 km distance to minimize contamination as much as possible and ensure participants will be exposed to the intervention(s);
- regular supermarket format (i.e., no compact store size);
- sufficient number of potential participants living in the supermarket’s neighbourhood (based on national data of *Statistics Netherlands,* the estimated number of inhabitants who are ≥45 years old and have a practical vocational education level is > 1600).
- a motivated supermarket manager showing engagement with the project and willingness to accept any randomisation outcome beforehand (based on qualitative interviews).

### Interventions

All intervention components were carefully developed and pre-tested based upon the needs, characteristics and preferences of the target population. We therefore have conducted a systematic review on healthy food nudges (PROSPERO registration number CRD42018086983) and investigated the preferences of the target population via qualitative interviews (manuscript in preparation). Also, we conducted a virtual supermarket experiment (Dutch Trial Register ID NL7095) testing the effect of various types of pricing strategies and salience nudges (manuscript submitted).

**Fig. 2:**
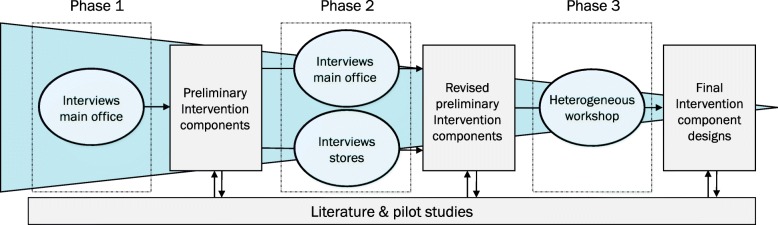
Co-creative process of the supermarket intervention components development

In another systematic review we have summarized factors that impede or enhance supermarket intervention implementation [56]. Next, development of the supermarket interventions involved the supermarket stakeholders and followed a co-creative process consisting of three phases (Fig. 2). The co-creative process was designed around cycles of increasingly defined ideas, discussion, and revision, following Participatory Action Research principles [57]. In phase one we drafted a preliminary set of nudging and pricing components based on interviews with Coop employees, literature reviews and pilot studies. Through interviews with managers and owners of Coop stores and key decision makers at the headquarters these components were further defined in phase two, and at times adjusted or dropped. In the third phase, a heterogeneous group of Coop stakeholders (including supermarket managers and employees of the headquarters with various types of positions within the organisation) ranked the revised components on perceived feasibility and expected outcomes, as such co-designing the final intervention components.

Nudging and pricing strategies will be targeted at promoting all healthy food groups in accordance with the Dutch Dietary Guidelines (**Table****S1**) [58], focussing on fruits and vegetables, legumes, grain products, fats, nuts, fish, drinks and dairy products. Both the nudging and pricing strategies aim to achieve a shift towards a healthier dietary pattern. Therefore, higher purchases of fruit and vegetables will be promoted and within all other food groups healthier options will be stimulated as substitutions of unhealthy options. Stimulating substitutions is not only crucial when aiming to sustainable change dietary behaviours, but also because merely promoting healthy choices can lead to both an increased purchase of healthy products, as well as an increased energy consumption, more food waste and spending of saved money (as a result of the subsidies on healthy products) on unhealthy products.

#### Healthy food nudges

A set of healthy food nudges will be implemented throughout supermarket sections containing healthy foods. We categorized the nudges into three nudging groups (**Table****S2**) derived from the typology of interventions in proximal physical micro-environments (‘TIPPME’) as proposed by Hollands et al. (2017) [59]: i) position and availability, ii) presentation and information; and iii) functionality. Position and availability nudges focus on product placement, altering proximity and access to foods by adding or removing products from a specific location, for example with healthy foods at the check-out counter. Presentation and information nudges use symbols, descriptions and pictures that highlight specific information about a food product combined with improvement of visual attractiveness of a product, such as via ‘shelf-talkers’ designed to grab attention. Considering the identified preferences of our target population, information nudges focus on product taste and convenience rather than on health. Last, functionality nudges guide product choice by providing positive feedback on food choices.

#### Pricing strategies

Price reductions of 25% will be implemented on healthy food groups (e.g., fruits and vegetables) and, whenever possible, price differences of 25% between unhealthy products and their healthy substitutes within the same food group (e.g., white bread and whole-grain bread) will be created. In order to do so, we will use a simultaneous price reduction (subsidy) of 15% on healthy products and a price increase (tax) of 10% on unhealthy products. Literature indicates that effectiveness of pricing strategies starts at 10–20% price changes, showing larger effects when combining taxes and subsidies [18, 34]. Following the co-creation process, supermarket stakeholders agreed upon price changes up to a maximum of 25%, with maximum price increases of 10%. Price increases will not be actively highlighted, but only communicated via the regular price shelf-tags. Price reductions will be highlighted using product promotion shelf-talkers and advertisements in the weekly door-to-door catalogues.

#### Implementation in the supermarkets

We will closely plan and monitor which foods will be targeted, as targeted products will be alternated over the course of the intervention to take into account regular supermarket promotions and (seasonal) variation in food product availability, to promote variation within a diet and to maximize intervention effects. With regard to the pricing strategies, the targeted product groups within the food group categories will alternate approximately every two to eight weeks depending on the regular Coop price promotions within a food group. With regard to nudging, some fixed nudges will be applied for the full intervention period (e.g., signage on shelf-tags) while others (e.g., end of aisle promotions) will vary every few weeks following a similar approach as for the pricing strategies.

#### Implementation monitoring and evaluation

The implementation process described above will be monitored and evaluated following validated Reflexive Monitoring in Action methods throughout the trial [60]. This interactive evaluation strategy facilitates implementation and provides insight on real-life barriers and facilitators encountered in a trial, to be used for the development of a roadmap for up-scaling.

We combine quantitative and qualitative methods. Throughout the trial, intervention stores will be visited by the research team and rated on implementation fidelity. Initially, monitoring visits will be bi-weekly, shifting towards monthly as the trial progresses. To score the implementation fidelity, a standardised checklist will be developed on which all individual components of both supermarket interventions can be rated on three-point Likert Scales with scores ranging from not implemented at all to implemented according to protocol (**Table****S3**). The intervention supermarket with the highest implementation scores after completion of the trial will receive an incentive, such as tickets for a team outing. Throughout the trial, a researcher will discuss the findings with the store manager and other relevant employees. These interviews aim to explore the underlying reasons for high and low fidelity scores, staff satisfaction regarding the implemented interventions, and seek solutions or useful strategies for (future) implementation of the interventions. Insights from these interviews are shared within the research team, and with other store managers, in an effort to secure engagement and learn from each other’s experiences – making the process reflexive. Based on these lessons, specifics parts of the implementation process for intervention components might be adjusted to optimize feasibility and fidelity.

We will allow minor deviations in implementation between supermarkets since store sizes and store layouts differ. Examples of minor deviations could be the exact placement of a shelf-talker nudge, or the total number of aisle baskets filled with healthy foods. If the monitoring system indicates major deviations in implementation fidelity between supermarkets during the trial, we will make sure to assist the supermarket with implementation according to protocol. If implementation according to protocol seems unfeasible, the nudging or pricing intervention may be reduced in dose, focused on different food categories or placed in a different position in the store. An adapted form will be implemented in all stores receiving the intervention(s). All adjustments will be documented. Documentation of intervention fidelity may inform the selection for per-protocol sensitivity analyses and used in developing the roadmap.

#### Mobile physical activity coaching app

A step counter app – suitable for both iPhone and Android devices – will be installed on the mobile phones of all participants randomised in the mobile coaching app intervention. Participants in the intervention group will additionally receive a smartphone-based PA coaching app aiming to increase the total daily step count, by providing just-in-time, personalised and context-specific walking tips throughout the day. The mobile PA app is based on the Telegram Messenger chatbot interface. It individually tailors message content by integrating participant baseline information, like preferences for coaching strategies and walking goals, with information stored on the mobile phone (current step count and proximity to location-based walking opportunities using geo-fencing techniques) and on the coaching server (responses to previous coaching attempts).

The intervention group will receive the personalized coaching messages via push notifications on their smartphone at crucial and contextual decision-making moments (e.g., choosing to take a five-minute detour through a nearby park to meet one’s daily step count goal). Advice will be selected from an existing database of messages [61], adapted to be readable and understandable by individuals with a low SEP (i.e., Dutch reading level B1). Depending on personal goals, current location, and responsiveness to previous similar messages, we will manually rank the most optimal messages on a day-to-day basis. Taking into account the number of steps already taken and the most suitable advice for that location, messages with the greatest chance of stimulating walking behaviour at that moment will be selected. When participants have used the app for a longer period of time, the selection and timing of the messages will be further adapted via machine learning techniques [62].

### Outcomes

#### Primary outcomes

The supermarket interventions are the primary focus of the trial, and therefore, the effects will primarily be evaluated based on the nudging and pricing interventions. The primary outcomes include the between-group differences in mean individual changes in three parameters of cardiometabolic health, namely systolic blood pressure, low-density lipoprotein (LDL) cholesterol and glycated haemoglobin (HbA1c), over 12 months compared with the control supermarkets. An additional primary outcome includes changes in dietary intake defined as changes on the Dutch Healthy Diet 2015(DHD15)-index as an indicator for adherence to the Dutch Dietary Guidelines [63], over 12 months compared with the control supermarkets as well.

#### Secondary outcomes

The following other cardiometabolic outcomes will be included as secondary outcomes: diastolic blood pressure, high-density lipoprotein (HDL) cholesterol, total cholesterol (TC), TC/HDL-ratio, triglycerides (TG) and waist circumference. Walking behaviours (i.e., step count) will be assessed to evaluate effects of the mobile PA app. In addition, we will assess intermediate behavioural factors including changes in food purchasing in the supermarket, food decision styles, social cognitive factors in relation to nudges and walking behaviours, customer satisfaction over 12 months, and acceptance of nudges and technology at 12 months, all compared with the control arm.

#### Roadmap

Following the previously described [54] approach for applying System Innovation and Transition Management theories to define the best strategies for implementation and upscaling beyond the study period we will use the reflexive monitoring of this trial as part of the co-creation of the final roadmap. This strategic roadmap will be based on gathered evidence on underlying mechanisms and structures and will include identified systemic barriers and facilitators, as well as strategies to overcome identified barriers for future supermarket-based health interventions.

### Sample size

We aim to recruit a total sample of 1485 participants, i.e., 495 participants per supermarket arm and 165 participants per supermarket location. With this sample size, the trial is powered to detect a mean difference of 3 mmHg in systolic blood pressure, 0.2 mmol/l in LDL-cholesterol and 0.2% in HbA1c, at 12 months, assuming the standard deviation for blood pressure is 12 mmHg, for LDL-cholesterol 0.85 mmol/l [6, 7, 64–67] and for HbA1c 1,05% [68–72]. These estimated effect sizes are at the lower range in comparison with results from previous lifestyle intervention trials, as we considered a small effect would be the most realistic to expect from our type of interventions. We expect a change of 5 points on the DHD15-index, assuming a standard deviation of 20 points [63]. With 80% power and a two-sided type 1 error rate of 0.05, the trial would require 283 participants in each supermarket arm to show this. We used a relatively small design factor of 1.4, as literature [73] and our estimations – based on cohort data from the Dutch new Hoorn Study [74] – shows intra-cluster correlations coefficients for CMD risk markers of below 0.01 within neighbourhoods, and allows for 25% drop out. As such, 495 participants need to be recruited per trial arm. With this sample size, the trial is powered to evaluate the effect of the mobile PA app as well, expecting a mean difference of 500 steps per day between the two groups, assuming a standard deviation of 2500 steps [75]. Again with 80% power and a two-sided type 1 error rate of 0.05, 392 participants should be included in the mobile PA app group. Allowing for 25% drop out, 980 participants from our total sample size of 1485 are required to be additionally randomised within the mobile PA app intervention.

### Recruitment

Various recruitment strategies will be combined to reach the targeted sample size. We will start with a number of passive strategies, including local advertisements in the study areas aiming to create awareness about the trial among potential participants. Strategies for local advertisement will be approaching local newspapers and online media with an interview request. In addition, we will ask supermarket cashiers to distribute flyers to every customer, display posters in-store and at other relevant locations (e.g., library, church, sport clubs, or community centres), post municipality targeted Facebook advertisements including short videos on the project, and send out postal invitations to every household of the municipality around the included supermarkets. Next, active recruitment strategies will be applied through community-outreach. Community-outreach methods will be tailored to the supermarket neighbourhood and its inhabitants, based on insights from the supermarket manager who is familiar with the customers, the neighbourhood and the community activities. In addition, we will search within every neighbourhood for important community-members who could facilitate community entry-points (e.g., public health services and community centres). Lastly, we will actively recruit in-store and during local events when available at time of recruitment.

Participant inclusion rates will be monitored per location with regard to sex and age distributions. We aim to include at least 30% males and at least 20% of participants aged 45–55 years, as we expect greater difficulty in recruiting those two groups. In order to meet those criteria, specifically targeted recruitment strategies will likely be needed. During baseline measurements, we will ask included participants if they know males and individuals aged 45–55 in their network, who are also regular shoppers of the selected supermarket and who would like to participate in the trial. To further boost the recruitment of male participants, female participants will be requested to encourage their male partner to register for screening. Participants will receive a small present (e.g., Coop merchandise products like a reusable water bottle and/or supermarket vouchers) after completion of each measurement moment, and a grocery box after completion of the last measurement. During the trial, participants will be provided with regular study updates and e-mail reminders prior to measurement appointments.

### Randomisation and allocation

The supermarket level and individual level randomisation sequences will be computer-generated using a variable block randomization tool in a web-based data management application (Castor Electronic Data Capture (EDC)). The nine supermarkets will be equally randomised in blocks of three, over the two store intervention arms and the control arm. Participants will not actively be informed on the intervention allocation of their supermarket. However, due to the nature of the interventions, blinding of participants will not be possible.

All recruited participants that meet the inclusion criteria for being enrolled within the mobile PA app intervention will be equally randomised via block randomisation as well to either the mobile PA app or the control app. The researcher performing the baseline measurement will reveal and communicate the participants’ allocation – which will be concealed up until that moment within CastorEDC – in order to be able to install the applicable app(s) on the participants’ smartphone.

### Study timeline

The intervention phase of 12 consecutive months accounts for seasonal variation in shopping and PA behaviours and allows for measurement of long-term effects. Data on cardiometabolic risk markers will be collected at baseline, halfway follow-up at 6 months and at the end of follow-up at 12 months. Data on intermediate behavioural factors will also be collected at 6 and 12 months, with an additional short-term follow-up measurement at 3 months (Table 1).

**Table 1:** Study timeline

	STUDY PERIOD				
TIMEPOINT	Enrolment	T_0_ (baseline)	T_1_ (3 months)	T_2_ (6 months)	T_3_ (12 months)
**ENROLMENT**					
Eligibility screening	●				
Informed consent	●				
Allocation to mobile PA app		●			
**INTERVENTIONS**					
Control supermarkets		●	●	●	●
Supermarkets with nudges		●	●	●	●
Supermarkets with nudges and pricing strategies		●	●	●	●
Participants with control app		●	●	●	●
Participants with mobile PA app		●	●	●	●
**ASSESSMENTS**					
**Population characteristics**					
Age		●			
Sex		●			
Household size		●			
Smoking status		●			
Medical history		●			
Medication use		●	●	●	●
**Primary outcome**					
Systolic blood pressure		●		●	●
LDL-cholesterol		●		●	●
HbA1c		●		●	●
Healthy dietary intake		●	●	●	●
**Secondary outcomes**					
Diastolic blood pressure		●		●	●
HDL-cholesterol		●		●	●
Total cholesterol		●		●	●
Total cholesterol/HDL-ratio		●		●	●
Triglycerides		●		●	●
Waist circumference		●		●	●
Walking behaviour		●	●	●	●
Healthy food purchases		●	●	●	●
Food decision styles		●	●	●	●
Nudges and social cognitive factors		●	●	●	●
Walking behaviour and social cognitive factors		●	●	●	●
Customer satisfaction		●	●	●	●
Technology acceptance					●
Acceptance of nudges					●
**Covariates**					
Self-control		●			
Digital health literacy		●			
Food-related behaviours		●			
Price awareness and perception		●			
Supermarket proximity		●			
Shopping style		●	●	●	●
Shopping at other supermarkets		●	●	●	●

LDL: low density lipoprotein, HDL: high density lipoprotein, HbA1c: glycated haemoglobin, PA: physical activity

The first participant recruitment and screening phase is planned to start mid-2020, where recruitment and baseline measurements will be initiated simultaneously. To secure adequate time for participant recruitment and in order to make the data collection for the baseline measurements feasible, a staggered implementation approach will be used. First, we will start the trial in three supermarkets (one randomised to each condition), the next three will start a month later, and the last three another month later. All groups of three supermarkets will consist of a nudging supermarket, a nudging and pricing supermarket, and a control supermarket. We expect to complete data collection by the end of 2021.

### Procedures and data collection methods

#### Population characteristics

Information on population characteristics will be collected via an online baseline questionnaire, or via a mailed version when preferred by the participant. The first part of the baseline questionnaire collects data on sex (male, female), age (years), household size (number of adults, number of children < 21 years), smoking status (current, former, or never), and on the medical history defined as the presence of cardiovascular diseases, hypertension, hyperlipidaemia, and/or diabetes (yes, no, do not know). At baseline and during all follow-up measurements, use of antihypertensive, blood lipid lowering and/or oral diabetes medication or insulin therapy (yes, no, do not know) will be registered.

#### Cardiometabolic outcomes

Participants will be invited for the cardiometabolic risk markers measurement at a location within the neighbourhood of selected supermarkets (e.g., community centre). Measurements will be performed by trained research staff. Results will be directly reported in case report form using CastorEDC and orally communicated and documented for interested participants. When the measurements reveal undiagnosed CMD risk, participants will be informed immediately and referred to their general practitioner.

Blood pressure will be measured in mmHg and all measurements will be performed twice on the left arm of which the mean value will be calculated. If the difference between both measurements will be more than 10 mmHg in systolic blood pressure or more than 5 mmHg in diastolic blood pressure, measurements will be repeated until two consecutive measurements do not differ more than these criteria – with a maximum of four measurements in total. When there appears no agreement between consecutive measurements by measurement four, the average of the last two measurements will be calculated and reported. Measurements will be performed with the Welch Allyn NIBP 3400, which has an estimated mean measurement error of ±5 mmHg (standard deviation 8 mmHg) [76].

The blood lipid profile and HbA1c concentrations will be measured in mmol/L by a point-of-care (POC) testing device (model Cobas B 101). Two drops of blood will be obtained by one finger prick (19 μL for the blood lipid test and 2 μL for the HbA1c test). Blood samples will be collected non-fasted [77], and results will be immediately analysed and available within 6 min. Once the blood sample is analysed, the test cannot be reopened, re-analysed or stored, and will therefore be disposed. The lipid test directly measures TC, HDL-cholesterol and TG, out of which the TC/HDL-ratio and the LDL-cholesterol value will be calculated. The latter is estimated following the Friedewald eq. (LDL = TC - HDL - TG/5 measured in mg/dL) [77]. When the TG concentration exceeds 4.52 mmol/L, the calculated LDL-cholesterol will not be reported by the POC device since the Friedewald equation is no longer valid above these concentrations. A reliability study compared Cobas B 101 analysis to capillary whole blood analysis [78]. The Pearson Correlation coefficients for the TC and TG measurement were 0.99, and for HDL and HbA1c this was 0.98.

Waist circumference will be measured in cm and measurements will take place at the midpoint between the lower margin of the least palpable rib and the top of the iliac crest using a stretch-resistant measuring tape. The average of both measurements will be calculated if they are within a 1 cm range. If the difference exceeds 1 cm, both measurements will be repeated once and the average of the last two measurements will be calculated and reported. For this waist circumference measurement procedure, an intra-rater error of 1.3 cm and inter-rater error of 1.6 cm were observed [79].

#### Healthy dietary intake

Changes in dietary intake will be defined as mean individual change in DHD15-index scores [63]. This index is a measure of diet quality, assessing adherence to the Dutch dietary guidelines. The index consists of fifteen components representing the fifteen food-based Dutch dietary guidelines of 2015. Per component participants can be assigned with a score between 0 and 10, resulting in a total score between 0 (no adherence) and 150 (complete adherence). To demonstrate, the intake of 0 g of vegetables would score 0 points in the vegetable category, and an intake of > 200 g would obtain the maximum score of 10 points.

The Dutch Healthy Diet 2015 food frequency questionnaire (DHD15-FFQ) will be used to measure DHD15-index scores [80]. The DHD15-FFQ consists of 40 multiple choice questionnaire items asking participants on their food consumption during the previous month, covering all components within the Dutch dietary guidelines of 2015. These components include: vegetables, fruit, cereals, legumes, nuts, dairy, fish, tea, coffee, fats, red meat, processed meat, sugary drinks, salt and alcohol. Responses will be scored using the DHD15-index, ranging between 0 (no agreement with the guidelines) and 10 (full agreement with the guidelines) for each component. In addition, ‘unhealthy choices’ is added as an extra component to assess the intake of products outside of the dietary guidelines. The scores for all 15 components will be summed to one overall DHD15-index score.

The validity of the DHD-FFQ was investigated based on the previous version of the tool, which estimated the DHD-index based on the Dutch dietary guidelines of 2006 [80]. DHD-index scores based on DHD-FFQ were acceptably correlated with DHD-index scores based on a 180-item FFQ combined with a 24-h urinary sodium excretion value (Spearman’s correlations 0.56, 95% CI 0.52–0.60). Derived from the same reference method, the DHD-FFQ had a small mean overestimation of 3.6 points on the DHD-index.

#### Walking behaviour

Step count will be used as a proxy for walking behaviour, which will be objectively measured by the phone’s internal step counter app. During each measurement moment, the average step count of that particular week will be calculated and used as outcome measure. During the baseline measurement of CMD risk markers, participants will be assisted by a researcher to install the app(s) on their smartphone. All participants will be instructed to keep their phone on their body; preferably in the pocket of their trousers. The step counter app stores the number of steps and synchronizes this data with a secured server linked to the app. The number of times a user will be in close proximity of a predefined type of significant walking activity location (without storing their specific GPS locations), step count per time unit, and relevant app usage events (e.g., the number and type of received messages), will be stored as well. The system is designed in such a way that the GPS locations themselves are neither sent nor stored. The apps will be sent for approval to the Apple Store and Google Play store, and to ensure compatibility throughout the trial the apps will be updated when needed.

#### Healthy food purchasing

Individual food purchase data will be collected via the Coop loyalty card, which will be handed out to all participants at baseline. The loyalty card is an existing program within the supermarket chain, which is used by costumers to receive exclusive loyalty card promotions and collect credits to spend in web shops. It is a personal card linked to the user enabling tracking of individual purchasing data. Participants will be asked to use their loyalty card at every supermarket visit throughout the year. Supermarket staff will be instructed to ask all costumers at check-out whether they have brought their loyalty card. Furthermore, the regular study updates will also include reminders to use the loyalty card.

The outcome for healthy food purchasing is defined as changes between the four measurement moments in total healthy food purchases (percentage) relative to total food purchase, based on the number of single purchased food items per food product category, or in product grams when more appropriate (e.g., for product categories with high quantity of unpackaged foods such as fruits and vegetables). In addition, data on the date of purchase will be collected as well, to be able to evaluate seasonal effects. The number of supermarket visits during the study period can be derived from these data as an indicator for the exposure to the supermarket interventions.

#### Questionnaire data on secondary outcomes

An overview of all questionnaire items with regard to the secondary outcomes is presented in Table 2. Relevant questionnaire items were selected and adapted from existing questionnaires (see references in Table 2) to reduce participant burden and secure retention of our target population with a low SEP. The validity of the selected questionnaire items will be assessed by asking experts on the specific topics to confirm the relevance and comprehensiveness of the selected items. Next, we will assess the comprehensibility of all questionnaire items by pilot testing the questionnaire among the target group. Regarding the questionnaire items, participants will be asked to rate the items on seven-point Likert Scales (strongly disagree-strongly agree) unless indicated otherwise. Changes in reflective, habitual and impulsive food decision styles will be measured as these decision styles are expected to have important implications for the effectiveness of nudging and pricing strategies [100]. For example, it is possible that strong unhealthy purchase habits are challenging to influence through nudging [101], although it is still unknown whether nudges can form new habits in the long term [102, 103]. Questions from existing questionnaires are adopted to investigate three food related decision styles [81–85]. The same questionnaire will be used twice per measurement moment: first to investigate decision styles in relation to healthy products and second to investigate food decisions in relation to unhealthy products (*Fruit and vegetables* in Table 2 will be replaced with *Sweets and/ or cake*). Six questions from existing questionnaires are adopted to investigate changes in social cognitive factors and behavioural mechanisms which are expected to be influenced by nudging, investigating health goals, experienced convenience of healthy shopping, social norm, and attractiveness of healthy foods [86–91]. Similar as for the nudges, questions were also adopted from existing questionnaires to investigate social cognitive factors which are expected to be influenced by the mobile PA app, on changes in self-monitoring, planning of walking activities, social comparison, intention and self-efficacy [92–96]. Changes in customer satisfaction and appreciation of the supermarket environment will be investigated using adapted questions from customer satisfaction survey items of the supermarket chain. As the mobile PA app communicates via a technological system in a presumed conversational ‘human voice’ [104], we will also assess the perceptions of this conversational style [97]. The degree to which participants accept technology as an aid to enhance their level of PA will be assessed [98]. Participants will be asked if they have noticed any changes in the store [99], as it is assumed that transparency about nudging will not reduce its effectiveness [105, 106], and there might be stronger effects when participants are aware they are being nudged. Last, participants will be asked if they appreciate that the supermarket suggests product choices. All questionnaire data will be collected via the survey function in CastorEDC.

**Table 2:** Questionnaire items^a^ per secondary outcome including item references

Subject	Item	Reference
**FOOD DECISION STYLES**		
**Reflective**	*I compare different types of fruit and vegetables before I buy something*	[81]
	*I put fruit and vegetables on my shopping list in advance*	
	*I think carefully about what fruits and vegetables I will buy*	
	*I make a thoughtful choice for the fruit and vegetables that I buy*	
	*I choose my vegetables and fruit attentively*	
**Habitual**	*Buying fruit and vegetables is part of my routine*	[82, 83]
	*I always buy the same fruit and vegetables*	
	*I buy fruit and vegetables on autopilot mode*	
	*Buying fruit and vegetables is typically something for me*	
	*Buying fruit and vegetables is something I do by default*	
**Impulsive**	*I buy fruit and vegetables if I feel like it*	[81, 84, 85]
	*I buy fruit and vegetables spontaneously*	
	*I buy fruit and vegetables on a whim*	
	*I buy fruit and vegetables if it comes to mind*	
	*I buy fruit and vegetables when I see a special offer*	
**NUDGES AND SOCIAL COGNITIVE FACTORS**		
**Health goals**	*I think it’s important to eat healthy*	[86, 87]
**Healthy shopping**	*Healthy products are available in my supermarket*	[88, 89]
	*In my supermarket it is easy to do healthy shopping*	
**Perceived social norm**	*Others in my supermarket buy healthy products*	[90]
	*My friends and family eat healthy*	
**Attractiveness healthy foods**	*Healthy products are tasty*	[91]
**WALKING BEHAVIOURS AND SOCIAL COGNITIVE FACTORS**		
**Self-monitoring**	*In the last four weeks I have kept in my mind whether I have …*	[92]
	*… walked long enough*	
	*… walked often enough*	
	*… took sufficient steps by going walking*	
**Planning**	*In the last four weeks I knew before I went walking …*	[93]
	*… where I went for a walk,*	
	*… why I went for a walk*	
	*… when I went for a walk*	
	*… how long I went for a walk*	
	*… who I went for a walk with*	
**Social comparison**	*When thinking about people who are similar to you, do you think you walk less or more per day than those people? (much less than others-much more than others)*	[94]
	*How often do you compare your own walking behaviour with that of people who are similar to you (always – never)*	
**Self-efficacy**	*How certain are you at the moment that you could meet your daily walking goal even if … (not sure at all-very sure)*	[95, 96]
	*… I am tired*	
	*… I have a bad mood*	
	*… I have very little time available*	
	*…I am on vacation*	
	*… it is raining outside*	
**Intention**	*I intend to meet my daily walking goal every day of the week (yes, definitely – no, definitely not)*	
	*I am sure I will meet my daily walking goal every day of the week (yes, definitely – no, definitely not)*	
	*I plan to meet my daily walking goal every day of the week (yes, definitely – no, definitely not)*	
**CUSTOMER SATISFACTION**		
	*How satisfied are you with your Coop supermarket in general (very unsatisfied-very satisfied)*	
	*To what extent are you satisfied with... (very unsatisfied-very satisfied)*	
	*…the supermarket environment and atmosphere?*	
	*…the supermarket layout and routing?*	
	*...the supermarket tidiness?*	
	*…the assortment of food products?*	
	*…the general product prices?*	
	*...the product discount prices?*	
	*…the fruit and vegetable prices?*	
	*…the bread prices?*	
**TECHNOLOGY ACCEPTANCE**		
**Communication**	*The coaching app…*	[97]
	*...invites me for a conversation*	
	*...is open to a conversation with me*	
	*…uses conversation style communication with me*	
	*…tries to communicate with me in a human voice*	
	*…tries to make communication interesting for me*	
	*…tries to make communication with me fun*	
	*…would admit an error to me*	
	*…treats me as a human being*	
	*How the user interface of the coaching app works is...*	
	*…easy for me to learn*	
	*…clear for me to understand*	
	*…easy for me to understand*	
**Technology as aid to enhance physical activity**	*The step counter app is...*	[98]
	*…useful for me to keep track of my progress towards my daily walking goal*	
	*…valuable for me to keep track of my progress towards my daily walking goal*	
	*…good for me to keep progressing towards my daily walking goal*	
	*…helpful for me to meet my daily walking goal*	
**ACCEPTANCE OF NUDGES**		
**Nudge awareness**	*Have you noticed anything in the supermarket in the past 12 months? (yes/no)*	[99]
	*If so, what have you noticed? (free text)*	
**Appreciation**	*It is nice when the supermarket suggests choices to me*	

^a^Participants are asked to rate the items on seven-point Likert Scales (strongly disagree-strongly agree), unless indicated otherwise

### Measurement of covariates

#### Self-control

Levels of perceived general self-control will be measured once at baseline with an adapted version of the Cantril’s ladder [107]. The original version measures life satisfaction, for which also other subjective well-being measures outcome components were investigated showing good reliability and convergent validity [108]. Based on this, we have adapted the scale to measure experienced self-control. Having lower to moderate levels of experienced self-control might explain why nudges and/or pricing strategies might be more effective for some participants as compared to others [109]. A picture of a ladder will be displayed accompanied by the following text: *Imagine that this ladder shows how much discipline a person can have. The top of the ladder represents the most discipline, and the bottom the least discipline. Indicate on this ladder how high your level of discipline in general is*. The item will be rated on a ten-point Likert Scale (having absolutely no discipline to having the most discipline you can have).

#### Digital health literacy

The eHealth literacy scale will be used to investigate digital health literacy at baseline [110], considering the possible negative influence of low digital health literacy on the effectiveness of the mobile PA app intervention. The questionnaire consists of one main question, answered by 8 items. Items will be rated on a seven-point Likert Scales (strongly disagree-strongly agree). The main question asks *When it comes to finding information about walking on a smartphone, such as finding a walking route or information about the preferred number of steps per day, then (I know)* …, answered by for example … *which information can be found,* or … *I can distinguish between high and low quality information*. The validity study of the eHealth literacy scale showed sufficient internal consistency (alpha = .93) [111].

#### Food-related behaviours

It is possible that the supermarket interventions have a stronger effect among participants interested in grocery shopping or cooking. Therefore, healthy shopping, food preparation, meal and eating behaviours will be measured at baseline by a 39-item questionnaire as proposed by Crawford et al. [112]. Some examples of questions are *I plan meals before I go shopping, I enjoy cooking meals,* or *My family/household eats dinner together at a dinner table.* Participants will be asked to rate these questions on seven-point Likert scales (never-always). Higher item scores were associated with higher fruit and vegetable intake (estimated with odds ratios per single item [112]).

#### Price awareness and perception

Pricing strategies will likely be more effective in price aware participants compared to price unaware participants. Therefore, the questionnaire by Lichtenstein et al. [113] will be used to measure baseline price awareness and perception. We will investigate the items related to the three constructs price consciousness (5 items), sale proneness (6 items), and value consciousness (7 items) which were all identified via confirmatory factor analysis [113]. All items will be rated on seven-point Likert scales (strongly disagree-strongly agree). Examples of items are *I am not willing to go to extra effort to find lower prices* (price consciousness)*, If a product is on sale, that can be a reason for me to buy it* (sale proneness) and *I am very concerned about low prices, but I am equally concerned about product quality* (value consciousness).

#### Supermarket proximity

Data on participants’ postal code will be collected to estimate the distance between home address and supermarket as another measure of exposure. Furthermore, the data on postal codes will be used to investigate the broader food environment and identify potential risks for contamination.

#### Shopping style

Participants will be asked at baseline and during follow-up to report how many times per week they generally do their grocery shopping at one of the selected supermarkets, at which times of the day they usually do their groceries and how many minutes on average they spent in supermarkets. We expect these components to influence the exposure to the supermarket interventions.

#### Shopping at other stores

Information will be collected at baseline and during follow-up on the number of visits to other stores in order to investigate exposure to the interventions. Participants will be asked to answer the question *In the past two weeks I have (also) bought groceries from a …*. with the items *Another supermarket, Local market, Local farmer, Baker, Greengrocer, Fishmonger,* or *Butchery* (never, 1–2 time, 3–4 times, or 5 time or more)*.*

### Statistical methods

Analyses will be performed according to the intention-to-treat principle. Intra-cluster correlation coefficients will be calculated to explore the degree of clustering of participant data within supermarkets. To investigate intervention effects for each primary outcome parameter, a linear mixed model analysis will be applied. Analyses will be based on individual participant data with a random intercept at the subject level. Supermarkets will be added to the models as random intercept as well, to test clustering of individuals within supermarket locations and decision for improvement of the models will be determined based on the likelihood ratio test. When the model does not significantly improve, supermarket location will be included as covariate. All models will be adjusted for the baseline values of the outcome under consideration. Changes in the secondary outcomes (except steps per day and social cognitive determinants of walking) will all be analysed following a similar procedure as for the primary outcomes. Changes in (cognitive determinants of) walking behaviour will be investigated with linear mixed model analyses as well, but including supermarket clusters as a covariate considering the individual-level randomisation of the mobile PA app intervention related to these two outcomes. Missing data will not be imputed, since mixed models will use the maximum available data between and within subjects.

Additional exploratory analyses will be undertaken to assess whether the intervention effects are modified by sex or age. A multivariable model will investigate intervention effects adjusting for supermarket monitor scores (as indicator for implementation fidelity). Furthermore, if an inadequate balance in population characteristics between study arms will be observed after visual inspection, additional adjusted sensitivity analyses will be performed with adjustment for these imbalanced variable(s) to assess the robustness of the primary analysis. Path analysis will be used to examine mediation effects (whether the intervention components lead to lower CMD risk via the targeted behaviours, by investigating the magnitude and significance of paths), and interaction analysis to investigate moderation effects by covariates. In the case of deviations between intervention fidelity scores between supermarkets those will be used for per-protocol sensitivity analyses. Last, when trial results are in favourable direction towards lowering CMD risk markers, a health impact model will be used to predict the potential long-term impact of our intervention to reduce the population burden of CMD. This model will allow a quantitative estimate of our intervention on CMD incidence.

Data will be analysed with R statistical software using an appropriate multi-level analysis package and/or STATA software. All statistical tests and confidence intervals will be two-sided, and the *p*-value of < 0.05 will be considered statistically significant.

## Discussion

In the Supreme Nudge trial, we will address multiple current knowledge gaps. First, the single and combined effect(s) of healthy food nudges and pricing strategies – including price increases – in the supermarket will be evaluated on lifestyle behaviours and CMD risk markers in adults with a low SEP. Second, the effect of a mobile PA app that provides just-in-time and context specific feedback tailored to our target group will be evaluated on walking behaviour. Last, we will explore the contextual and systemic actors and factors relevant to the implementation of the supermarket interventions.

Some challenges of conducting this trial are anticipated. First, recruitment and retention of a population with a low SEP is known to be challenging [114]. However, the combination of multiple passive and active recruitment strategies, involving community key-members and inviting included participants to recruit others in their personal network are promising approaches to reach the targeted sample size. Providing incentives such as supermarket vouchers and regular study updates and reminders are examples of strategies that will be used to enhance participant retention. Second, questionnaire data collection among a population with a low SEP may be complicated due to for example low literacy. Consequently, using previously validated questionnaires may increase attrition. To enhance comprehensibility of items, we have shortened some existing questionnaires and used simpler language which will be pilot tested among the target group. Third, blinding of participants, supermarket managers and research staff will not be possible due to the nature of the interventions. Nevertheless, we expect that a lack of blinding will not majorly affect our results, since the interventions target automatic rather than reflective decision processes, being aware of nudging seems not to affect its effectiveness [105, 106], and because we use a number of objectively measured outcomes. Fourth, accurate measurement of dietary intake remains challenging when using self-reported measures. It is known to be affected by random and systematic errors [115]. Yet the alternatives (e.g., supplementing with objective measures such as biomarkers) are costly and majorly increase participant burden – threatening trial retention. However, by measuring both purchasing behaviour as well as dietary consumption behaviour we will be able to explore to what extent changes in food purchases translate into changes in consumption. Fifth, detecting changes in individual CMD risk factors will be challenging considering the small expected effect sizes attributable to our interventions. However, we accounted for such small effect sizes in our power calculation. Moreover, the probability of modest individual changes should be translated to potential CMD benefit on a population level to identify clinical relevance, for which we can use the health impact modelling. Sixth, and last, partnering with a supermarket chain creates the opportunity for co-creation of the nudging and pricing strategies to secure feasibility of the intervention components in the retail setting. However, in a real-life setting like our study, the risk of negatively affecting supermarket performance may impede fidelity of our intervention. Although we will carefully monitor implementation of the intervention and discuss any issues with participating stores, we cannot completely exclude this possibility. Nevertheless, potential financial setbacks linked to participation in the trial have been accepted by the supermarket management in advance. The established collaboration with the supermarkets is deemed crucial in order to create opportunities for large scale implementation within the retail setting and secure sufficient external validity of study findings.

Findings from the Supreme Nudge trial can be used to guide future public health policies to reduce health inequalities within populations using an environmental approach. It will provide a novel contribution to the existing body of evidence on strategies using environmental interventions to promote healthy lifestyle behaviours, by combining multiple promising strategies and providing insight in CMD benefits in a low SEP population and how interventions can be successfully implemented.

## Supplementary information

**Additional file 1: **Tables with information on the food products to (not) be targeted in the supermarket (**Table S1**), on the types of healthy food nudges and examples of in-store use (**Table S2**), and the implementation fidelity checklist (**Table S3**).

## Data Availability

Data sharing is not applicable to this article as no datasets were generated or analysed during the current study.

## Abbreviations

CMD(s): Cardiometabolic disease(s)
DHD15-index: Dutch Healthy Diet index 2015
HbA1c: Glycated haemoglobin
HDL: High-density lipoprotein cholesterol
LDL: Low-density lipoprotein cholesterol
PA: Physical activity
SEP: Socioeconomic position
TC: Total cholesterol
TG: Triglycerides
