# Correction: Reducing cardiometabolic risk in adults with a low socioeconomic position: protocol of the Supreme Nudge parallel cluster-randomised controlled supermarket trial

**DOI:** 10.1186/s12937-022-00795-9

**Published:** 2022-06-28

**Authors:** Josine M. Stuber, Joreintje D. Mackenbach, Femke E. de Boer, Gert-Jan de Bruijn, Marleen Gillebaart, Marjolein C. Harbers, Jody C. Hoenink, Michel C. A. Klein, Cédric N. H. Middel, Yvonne T. van der Schouw, Tjerk Jan Schuitmaker-Warnaar, Elizabeth Velema, Anne L. Vos, Wilma E. Waterlander, Jeroen Lakerveld, Joline W. J. Beulens

**Affiliations:** 1 Department of Epidemiology and Data Science, Amsterdam Public Health Research Institute, Amsterdam UMC, VU University Amsterdam, De Boelelaan 1089a, 1081 HV Amsterdam, the Netherlands; 2 Upstream Team, www.upstreamteam.nl, Amsterdam UMC, VU University Amsterdam, Amsterdam, the Netherlands; 3 Department of Social, Health and Organizational Psychology, Utrecht University, Utrecht, the Netherlands; 4 Department of Communication Science, University of Antwerp, St-Jacobstraat 2, 2000 Antwerpen, Belgium; 5 Julius Center for Health Sciences and Primary Care, University Medical Center Utrecht, Utrecht University, Utrecht, the Netherlands; 6 Centre for Diet and Activity Research, MRC Epidemiology Unit, University of Cambridge School of Clinical Medicine, Institute of Metabolic Science, Cambridge, CB2 0QQ UK; 7 Social AI Group, Department of Computer Science, VU University Amsterdam, Amsterdam, the Netherlands; 8 Athena Institute, Faculty of Science, VU University, Amsterdam, The Netherlands; 9 Netherlands Nutrition Centre (Voedingscentrum), The Hague, The Netherlands; 10 Amsterdam School of Communication Research ASCoR, University of Amsterdam, Amsterdam, the Netherlands; 11 Department of Public Health, Amsterdam Public Health Research Institute, Amsterdam UMC, University of Amsterdam, Amsterdam, the Netherlands

**Correction: Nutr J 19, 46 (2020)**

**https://doi.org/10.1186/s12937-020-00562-8**

This erratum describes changes made in our previously published study protocol [1], as the occurrence of the COVID-19 pandemic has led to insurmountable challenges in feasibility to maintain the original design. The planned start of participant recruitment for the trial coincided with the first COVID-19 lockdown in the Netherlands starting March 16 2020. As a result, the start of the study was postponed by 11 months. The continued/renewed lockdown hampered face-to-face contact and thus some planned physical measurements. These circumstances required adaptation to remote data collection methods, revision of recruitment goals and of the primary study outcomes, and inclusion of additional study sites to secure adequate participant inclusion rates. Therefore, below we present the revised methodology of the Supreme Nudge parallel cluster-randomised controlled supermarket trial. All changes made to the original protocol were reviewed and approved by the Medical Ethics Review Committee of VU University Medical Center (reference number: 2019.334) prior to implementation.

## Study design

As previously described [1], the Supreme Nudge supermarket trial is a cluster randomised controlled design, with nudging and pricing strategies implemented at the supermarket level (Fig. 1). The original protocol described two groups of intervention supermarkets (nudging or nudging and pricing) and one group of control supermarkets. This design is revised to two groups in total; supermarkets are randomized to an intervention group receiving the combination of healthy food nudges and pricing strategies or a control group receiving no intervention. The groups for the mobile physical activity (PA) coaching app remain unchanged; the coaching app is randomised at the individual level across all supermarket clusters.

**Fig. 1:**
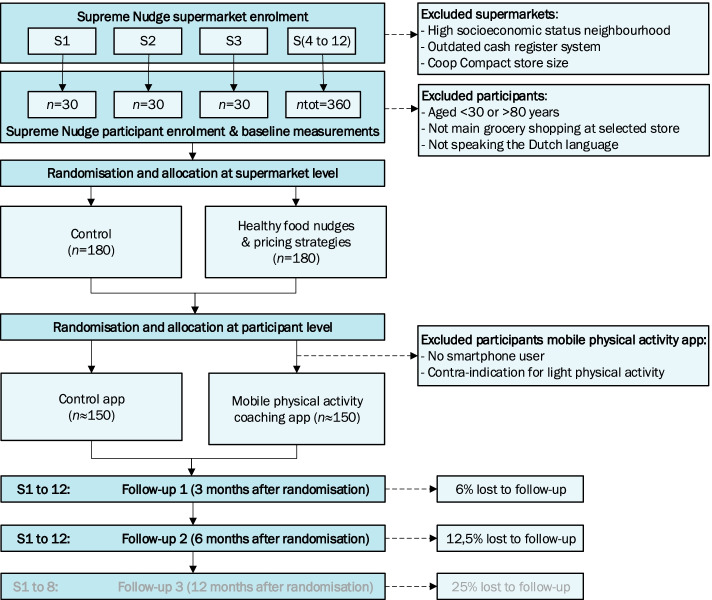
Revised design of the SUPREME NUDGE randomised controlled trial

The original protocol described inclusion of eight supermarkets and an intervention duration and follow-up time of 12 months [1]. However, recruitment among eight supermarkets led to insufficient participant enrolment, which necessitated additional inclusion of four additional supermarket locations. As a result, eight out of 12 supermarkets were enrolled within the study in the spring of 2021 and the four additional supermarket locations in the autumn of 2021. For these four stores, the intervention implementation and the follow-up time is only feasible for a duration of six months due to ending of project funding by the summer of 2022.

## Supermarkets

Twelve supermarkets of a Dutch supermarket chain that meet all of the following criteria are included in the study:

i) Regular supermarket format, i.e., no compact store size (unchanged criterion);
ii) Located in a lower SEP neighbourhood (below average postal code SEP-scores of The Netherlands Institute for Social Research [2]) (unchanged criterion);
iii) Implemented a new cash register system which allowed implementation of pricing strategies (new criterion).

## Participants

Participants are men and women living in a low SEP neighbourhood. Used inclusion criteria are:

i) Aged 30–80 years (was 45–75 years in the original protocol);
ii) Living in a lower SEP neighbourhood surrounding one of the participating supermarkets (was combined with having a practical vocational education level in the original protocol);
iii) Self-report to do (or report their partner does) more than half of the household grocery shopping at the selected supermarket and plan to continue visiting for the next (half) year (unchanged criterion);
iv) Provide written informed consent (unchanged criterion);
v) Able to communicate in the Dutch language (unchanged criterion).

## Trial outcomes

We revised our primary outcomes to changes in the dietary intake (DHD15-index scores [3]) over 6 or 12 months for all intervention supermarket participants compared to control participants. Secondary outcomes now include cardiometabolic outcomes, including HbA1c, low-density lipoprotein (LDL) cholesterol, high-density lipoprotein (HDL) cholesterol, total cholesterol (TC), TC/HDL-ratio, triglycerides (TG) and waist circumference. The planned blood pressure measurement was excluded due to feasibility limitations when we needed to adapt to remote data collecting methods. Regarding the other previously described secondary outcomes, some questionnaire items relating to walking behaviours and social cognitive factors have been revised. Furthermore, we added healthy dietary intake per food group based on the sub-scores of the DHD15-index scores [3], items on self-reported walking behaviour, use of the coaching app and walking app, privacy concerns relating to the coaching app and on perceptions on healthy eating and on grocery shopping (described in detail below).

## Sample size

The current set-up of the trial is powered to detect a mean change of 5 points on the DHD15-index, assuming a 15 point standard deviation of the mean change. This standard deviation is based on the first round of collected baseline data in the spring of 2021, where the standard deviation of the DHD15-index score was 19.9. To estimate a standard deviation around the mean difference, we used the formula σ √[2(1-ρ)], assuming a ρ of 0.75, resulting in a standard deviation of the mean difference of 14.1. With 80% power and a two-sided type 1 error rate of 0.05, the trial required 141 participants in each supermarket arm. To allow for 25% drop out, a minimum of 176 participants need to be recruited per arm, resulting in 352 participants in total. We set our recruitment goal at 360 participants, resulting in a mean of 30 participants per cluster (i.e., supermarket). We did not use a design factor, as our estimations based on our first round of baseline data showed no correlation for observations of DHD15-index scores between study sites.

## Recruitment

As planned, a stepwise recruitment strategy was applied. However, active recruitment possibilities were limited due to the COVID-19 lockdown and planned neighbourhood tailored community-outreach methods and active recruitment at local events were not possible. As alternative active recruitment method participants were requested via a phone call to encourage their partner or neighbours to register for eligibility screening (promoting word-of-mouth). Recruiting in-store by the research team was conducted as soon as COVID-19 related restrictions allowed, which started approximately halfway through the recruitment period. Furthermore, additional passive recruitment strategies were applied throughout the whole recruitment period. The initial planned passive strategies started with local advertisements including news articles in local (online) media, flyers distributed in the supermarket and by mail, posters displayed in-store and at some other locations in the neighbourhood (e.g., physiotherapy practice), postal invitation letters sent to every household of the municipality around the included supermarkets, and municipality targeted Facebook advertisements. The additional passive strategies were an email invitation to the supermarket’s customer panels, Facebook posts on the participating supermarket pages, and advertisement on the website of the Dutch Heart Foundation (study funder).

## Study procedures

All data collection procedures are adapted towards remote data collection methods, instead of inviting participants to a study location. The eligibility screening and inclusion procedure was as follows: Interested supermarket customers registered via the project website, by telephone, or mailing a register form which was included on the supermarket flyer. Next, they received an online screening questionnaire including all items regarding the inclusion and exclusions criteria. Those who appeared eligible based on the screening received a mail with participant information form and consent from and a free of charge reply envelope. Researchers called all eligible participants two or three days after sending the information form to elaborate on potential participation and to provide the opportunity the ask questions. Next, eligible participants were invited to sign the consent form and return it in the return envelope.

Baseline data collection started as soon as the researchers received the completed consent form. Participants were sent an email explaining study procedures regarding the at-home-measurement kit for the cardiometabolic measurements, the online questionnaires, instructions for the use of a supermarket loyalty card, and, if applicable, instructions for downloading and installation of the step counter app and the coaching app. Instructions referenced a web-based step-by-step guide with pictures, including a video showing the same steps. On the same day of sending this email explaining study procedures, the at-home-measurement kit was sent to the participants home address by mail. The kit consisted of a blood test and a waist circumferences measuring tape, including an instruction letter referencing a web-based instruction video. Four to seven days after sending the at-home-measurement kit, participants received two web-based questionnaires. The first questionnaire asked about lifestyle factors and provided opportunity to fill in the results from the at-home waist circumference measurement. The second questionnaire concerned questions on the dietary intake (DHD15-index). Participants not using email received the questionnaires via mail, including a free of charge return envelope.

## Collected data

Table 1 presents an updated version of the study time line and included measurements and changes in collected data and questionnaire items are detailed below.

**Table 1:** Study timeline and collected data

TIMEPOINT	STUDY PERIOD				
	Enrolment	T_0_ (baseline)	T_1_ (3 months)	T_2_ (6 months)	(T_3_ (12 months))^a^
**ENROLMENT**					
Eligibility screening	●				
Informed consent	●				
Allocation to mobile PA app		●			
**INTERVENTIONS**					
Control supermarkets		●	●	●	(●)
Supermarkets with nudges and pricing strategies		●	●	●	(●)
Participants with control app		●	●	●	(●)
Participants with mobile PA app		●	●	●	(●)
**ASSESSMENTS**					
**Population characteristics**					
Age		●			
Sex		●			
Household size		●			
Smoking status		●			
Medical history		●			
Medication use		●	●	●	(●)
**Primary outcome**					
Healthy dietary intake		●	●	●	(●)
**Secondary outcomes**					
HbA1c		●		●	(●)
LDL-cholesterol		●		●	(●)
HDL-cholesterol		●		●	(●)
Total cholesterol		●		●	(●)
Total cholesterol/HDL-ratio		●		●	(●)
Triglycerides		●		●	(●)
Waist circumference		●		●	(●)
Healthy dietary intake per food group		●	●	●	(●)
Healthy food purchases		●	●	●	(●)
Food decision styles		●	●	●	(●)
Nudges and social cognitive factors		●	●	●	(●)
Customer satisfaction		●	●	●	(●)
Perceptions on healthy eating				●	(●)
Perceptions during grocery shopping				●	(●)
Acceptance of nudges				●	(●)
Walking behaviour		●	●	●	(●)
Walking behaviour and social cognitive factors		●	●	●	(●)
Communication coaching app			●	●	(●)
Use of coaching app			●	●	(●)
Technology acceptance coaching app			●	●	(●)
Privacy concerns coaching app				●	(●)
Self-reported walking behaviour				●	(●)
**Covariates**					
Self-control		●			
Digital health literacy		●	●	●	(●)
Food-related behaviours		●			
Price awareness and perception		●			
Supermarket proximity		●			
Shopping style		●	●	●	(●)
Shopping at other supermarkets		●	●	●	(●)

^a^ Measurements at month 12 are only performed for first the eight included supermarket locations which with enrolment in the spring of 2021

The blood test measures lipid profile and HbA1c concentrations using a finger prick, collecting capillary blood into two small capillary tubes which are sent back to the lab via a medical reply envelope in the mail. Blood samples are collected non-fasted by the participant at home, or if desired by the participant with help from trained research staff during a home visit. Four blood drops are obtained with a single finger prick for the HbA1c test tube, and 16 blood drips for the lipid test tube. Participants are instructed to put their blood sample back in the package designed for medical postal service and ship the test at day of collection. When the outside temperature is <4 or > 25 degrees of Celsius, the participant is instructed to drop the package at a postal office.

Blood samples collected via the at-home measurement kit are analysed by the Amsterdam UMC – VUmc clinical laboratory. HbA1c in the whole blood remains stable at room temperature and the at-home-measurement via the finger prick test of HbA1c is therefore a standard procedure used in the hospital. However, the at-home-measurement of blood lipids was not a standard procedure at study conception. Therefore, the stability of the lipid profile in whole blood kept at room temperature was pilot tested by the laboratory prior to the study. Tubes partly prefilled with heparin were used to secure preservation of the sample for blood plasma analysis. The pilot test was conducted by leaving ten blood samples at room temperature for seven days. Up to day three, the absolute mean change in LDL was deemed acceptable (15% change) as compared to the samples directly analysed at day zero (Table 2). All blood test results were analysed by enzymatic colorimetric test via the Roche/Hitachi Cobas C systems. The lipid test directly measures total cholesterol (TC), HDL-cholesterol and triglycerides (TG). Based on these measurements, the TC/HDL-ratio and the LDL-cholesterol value was calculated following the Friedewald equation. During the baseline measurements, 89% of blood samples were analysed at day 1 after collection, confirming the feasibility of this procedure.

**Table 2:** Absolute (mmol/L) and relative (%_change_) changes in lipid profile outcomes after storing the blood samples at room temperature from day 0 up to day 7 (n = 10)

	Cholesterol		HDL		LDL		Triglycerides		non-HDL		TC/HDL-ratio	
	mmol/L	%	mmol/L	%	mmol/L	%	mmol/L	%	mmol/L	%	Ratio	%
**Day 0**	4.09	1	1.57	1	1.83	1	1.54	1	2.52	1	2.79	1
**Day 1**	4.19	1.03	1.57	1.00	1.93	1.07	1.55	1.00	2.63	1.06	2.86	1.03
**Day 2**	4.29	1.05	1.61	1.02	1.98	1.12	1.55	1.00	2.69	1.08	2.86	1.03
**Day 3**	4.34	1.06	1.62	1.03	2.04	1.15	1.54	0.99	2.73	1.10	2.85	1.03
**Day 4**	4.40	1.08	1.63	1.05	2.09	1.20	1.52	0.98	2.77	1.13	2.86	1.04
**Day 5**	4.43	1.09	1.67	1.07	2.08	1.19	1.52	0.98	2.76	1.12	2.82	1.02
**Day 6**	4.46	1.10	1.68	1.07	2.10	1.20	1.52	0.98	2.79	1.13	2.83	1.02
**Day 7**	4.50	1.11	1.67	1.07	2.15	1.24	1.50	0.97	2.83	1.16	2.87	1.04

Table 3 shows a revised version of all questionnaire items related to secondary outcomes. We have added items relating to perceptions on healthy eating, perceptions during grocery shopping, walking behaviours and social cognitive factors, self-reported walking behaviour and privacy concerns relating to the coaching app. Regarding covariates measured, we have added a question whether participants have been pregnant in the past year considering the waist circumference measurement.

**Table 3:** Questionnaire items^a^ per secondary outcome including item references

Subject	Item	Reference
**FOOD DECISION STYLES**		
**Reflective**	*I compare different types of fruit and vegetables before I buy something.*	[4]
	*I put fruit and vegetables on my shopping list in advance.*	
	*I think carefully about what fruits and vegetables I will buy.*	
	*I make a thoughtful choice for the fruit and vegetables that I buy.*	
	*I choose my vegetables and fruit attentively.*	
**Habitual**	*Buying fruit and vegetables is part of my routine.*	[5, 6]
	*I always buy the same fruit and vegetables.*	
	*I buy fruit and vegetables on autopilot mode.*	
	*Buying fruit and vegetables is typically something for me.*	
	*Buying fruit and vegetables is something I do by default.*	
**Impulsive**	*I buy fruit and vegetables if I feel like it.*	[4, 7, 8]
	*I buy fruit and vegetables spontaneously.*	
	*I buy fruit and vegetables on a whim.*	
	*I buy fruit and vegetables if it comes to mind.*	
	*I buy fruit and vegetables when I see a special offer.*	
**NUDGES AND SOCIAL COGNITIVE FACTORS**		
**Health goals**	*I think it’s important to eat healthy.*	[9, 10]
**Healthy shopping**	*Healthy products are available in my supermarket.*	[11, 12]
	*In my supermarket it is easy to do healthy shopping.*	
**Perceived social norm**	*Others in my supermarket buy healthy products*	[13]
	*My friends and family eat healthy.*	
**Attractiveness healthy foods**	*Healthy products are tasty.*	[14]
**CUSTOMER SATISFACTION**	*How satisfied are you with your Coop supermarket in general (very unsatisfied-very satisfied)*	N/A
	*To what extent are you satisfied with... (very unsatisfied-very satisfied)*	
	*…the supermarket environment and atmosphere?*	
	*…the supermarket layout and routing?*	
	*...the supermarket tidiness?*	
	*…the assortment of food products?*	
	*…the general product prices?*	
	*...the product discount prices?*	
	*…the fruit and vegetable prices?*	
	*…the bread prices?*	
**PERCEPTIONS ON HEALTHY EATING**		
**Motivation to eat healthy**	*I want to eat healthy.*	N/A
**Reasons for heathy eating**	*The reason I want to eat healthy is…* *…because this is important for good health.* *…because it fits my life goals.* *…because I would feel bad about myself if I did not.* *…because I feel pressure from others to do this.*	[15]
**PERCEPTIONS DURING GROCERY SHOPPING**		
**Grocery decisions**	*What do you think about your groceries choices bought at your Coop supermarket?* *…I feel that my choices fit my personal preferences perfectly.* *…I feel that I have been able to influence my choices.* *…I am satisfied with my choices.* *…These were choices I could not make very well.*	[16]
**Shopping experience**	*How did you feel doing your shopping in your Coop supermarket?* *…I felt patronized when choosing my groceries.* *…I felt encouraged to choose healthy products.* *…I felt invited to choose healthy products.*	[17]
**Nudge awareness**	*Have you noticed anything in the supermarket in the past 12 months? (yes/no)*	[18]
	*If so, what have you noticed? (free text)*	
**Appreciation**	*I appreciate it when the supermarket helps me to make healthy choices.*	
**WALKING BEHAVIOURS AND SOCIAL COGNITIVE FACTORS**		
	*In the last four weeks (baseline)/ three months (follow-up 1)/ six months (follow-up 2 and 3),…*	[19, 20]
**Consequences of behaviour**	*...I have searched for and/or red information about the (health) benefits of walking*	
**Social comparison**	*...I noticed how much I walked compared to others.*	
**Action planning**	*...I planned in advance when and where I would go hiking.*	
**Self-monitoring**	*...I kept in mind whether I walked enough.*	
**Social support**	*..I have searched for support from others to walk enough.*	
**Goal setting**	*...I have set achievable (yet challenging) walking goals for myself.*	
**barrier identification**	*...I looked for tips on how to overcome barriers to walking (such as too little time).*	
**Self-evaluation**	*...I checked with myself how satisfied I am with my walking behaviour.*	
**Encouragement**	*...I looked for and/or read information that encouraged me to walk enough.*	
**Others’ approval**	*...I paid attention to what others think of my walking behaviour.*	
**COMMUNICATION**	*The coaching app…*	[21]
	*...invites me for a conversation*	
	*...is open to a conversation with me*	
	*…uses conversation style communication with me*	
	*…tries to communicate with me in a human voice*	
	*…tries to make communication interesting for me*	
	*…tries to make communication with me fun*	
	*…would admit an error to me*	
	*…treats me as a human being*	
	*...trying to force something on me* *…appears patronizing*	
**USE OF COACHING APP**	*In the past three months, have you used a subscription with a mobile internet data bundle on the smartphone on which the walking coach is installed? (yes/no)* *How much data could you use monthly within your mobile internet data bundle? (five categories: 1/5/10/more than 10 GB per month, or: I do not know)* *How often do you read the messages you receive from the walking coach? (never-always)* *Which functions or characteristics of the walking coach are you positive about? (free text)* *Which functions or characteristics of the walking coach are you negative about? (free text)* *Do you have any other comments about your experiences with the walking coach? (free text)*	N/A
**TECHNOLOGY ACCEPTANCE**		
**Perceived ease of use**	*How the walking app’s user environment works is…* *...easy for me to learn.* *...clear for me to understand.* *...easy for me to understand.*	[22]
**Perceived usefulness**	*The walking app…* *…is helpful for me to keep track of my daily walking goal.* *…is valuable to me to track progress towards my daily walking goal.* *…works well for me to keep working towards my daily walking goal.* *…is helpful to me in meeting my daily walking goal.*	[22]
**SELF-REPORTED WALKING BEHAVIOUR**	*Thinking about the past 4 weeks, on how many days did you walk for at least 10 minutes in a row?* *On the days when you walked for at least 10 minutes, how much time did you usually spend walking?*	[23]
**PRIVACY CONCERNS COACHING APP**	*Because I used the walking app…* *…I have the feeling that others know more about me than I would like.* *…I think others can see my private information.* *…I feel that my private information can be misused more easily.*	[24]

## Statistical analysis

Population baseline characteristics will be described stratified by trial arm, to examine adequate balance between intervention groups and to provide an overview of the study population. Baseline differences between intervention groups will be visually inspected to detect potential clinically relevant differences. Baseline characteristics will be summarised as mean and standard deviation for normally distributed continuous variables, and median and interquartile range for skewed continuous variables. Frequencies and percentages will be presented for each category of categorical variables.

Collected data on the DHD15-index scores will be treated as continuous outcome variables. To investigate intervention effects a multilevel analysis (i.e. linear mixed models) will be applied. Analyses will be based on individual participant data, with a random intercept at the subject level and potentially including a random slope for supermarket location. The outcome model will be adjusted for the baseline value in order to take into account the regression to the mean phenomenon. Changes in the secondary outcomes, including all cardiometabolic outcomes, the percentage of healthy food purchase, food decision styles, social cognitive factors, perceptions, and customer satisfaction, will all be treated as continuous outcome variables and analysed following a similar procedure as described at the multilevel analysis for the primary outcome.
