# Supplementary material for: Reducing cardiometabolic risk in adults with a low socioeconomic position: protocol of the Supreme Nudge parallel cluster-randomised controlled supermarket trial
Source: Nutr J. 2020 May 19;19:46. doi: 10.1186/s12937-020-00562-8 (PMC7236937; doi:10.1186/s12937-020-00562-8)
Supplement: Supplementary file 1 — Additional file 1: Tables with information on the food products to (not) be targeted in the supermarket (Table S1), on the types of healthy food nudges and examples of in-store use (Table S2), and the implementation fidelity checklist (Table S3). [file 12937_2020_562_MOESM1_ESM.docx]

**Table S1. Food products to (not) be targeted in the supermarket**

| ***Product category*** | ***Products to promote*** | ***Products NOT to promote/ products to discourage*** |
| --- | --- | --- |
| **Fruits and**  **vegetables** | - Fresh and pre-cut vegetables  - Frozen and canned vegetables without added sugar and salt  - Fresh and pre-cut fruit  - Frozen fruit without added sugar | - Canned vegetables with added sugar or salt  - Vegetable and fruit juice  - Vegetable a la crème  - Dried fruit  - Canned fruit |
| **Grain products** | - All whole grain products (e.g., (muesli)bread, crackers, noodles, pasta, couscous, rice, rye bread), with >5 grams of fibre per 100-gram product  - Breakfast cereals low in sugar, fat and salt (oatmeal, oat flakes, muesli) | - All refined grain products (e.g., (muesli)bread, crackers, croissant, noodles, pasta, couscous, rice, bulger), with <5 grams of fibre per 100-gram product  - Potato, ready-made mashed potatoes and mashed potato puree  - Breakfast cereals with added sugar, fat and salt (e.g., cruesli and corn flakes) |
| **Legumes** | - Legumes from canned or frozen  - Dried legumes | - Canned legumes with added sugar and salt (such as kidney beans in chili sauce or white beans in tomato sauce) |
| **Fats** | - Olive and sunflower oil  - Soft margarine or low-fat margarine for bread  - Liquid margarine and liquid frying fat | - Hard margarine, baking and frying fat and deep-frying fat  - Butter  - Coconut oil and palm oil |
| **Nuts** | - Nut butter and peanut butter of 100% nuts or peanuts  - Unsalted (pea)nuts | - Salted (pea)nuts  - Coated (pea)nuts  - (Pea)nuts with chocolate or sugar  - Nut paste and peanut butter with added salt, sugar and/or (palm) fat |
| **Fish** | - Fresh and frozen unprocessed fish  - Breaded fish (containing >70% fish)  - Shellfish | - Smoked / pickled fish  - Canned fish with added salt  - Breaded fish (containing <70% fish) |
| **Drinks** | - Water  - Black and green tea  - Herbal tea | - Beverages with sugar (100% fruit juice or vegetable juice, sweetened juices, soft drinks, syrup, juice, sweetened dairy drink, sports drink and energy drink)  - Alcoholic drinks  - Coffee  - Sugar-free soft drinks (artificial sweetened) |
| **Dairy** | - Skimmed and semi-skimmed milk, buttermilk  - Skimmed and semi-skimmed yogurt unsweetened  - Low-fat quark unsweetened  - Drink yoghurt unsweetened  - Soy drink with added vitamin B12 and calcium unsweetened  - Cheese low in fat (<14 gram saturated fat per 100 gram) and low in salt (<2 gram per 100 gram)  - Dairy spread  - Hüttenkäse  - Mozzarella  - Fresh goat cheese | - Dairy and soy drinks with added sugar  - Rice drink and almond drink  - Pudding and desserts with added sugar  - Custard  - Ice cream, soft ice cream and yoghurt ice cream  - 48+ and 60+ cheese  - Feta  - Crème fraîche, sour and cooking cream  - Whipped cream  - Whole-fat milk and yogurt products |

**Table S2. Types of healthy food nudges and in-store use examples**

| **Type nudge** | **Content nudge** | **Description nudge** |
| --- | --- | --- |
| **Position & availability** | **Check-out purchases** | Increase healthy food options or non-food at check-outs. |
|  | **End of aisle** | Healthy foods on end of aisle promotions. |
|  | **Baskets in the aisles** | Healthy products on 'decision endpoints' at the end of other shelves, or next to products with which it can be combined. |
|  | **Product shelf ratio and arrangement on eye height or closer up front** | Improving product visibility through adjustment shelf-product ratio and through eye-height positioning or placement closer/ further away (in low/high shelves). |
|  | **‘What are we eating today’ inspiration** | Promote healthy products at *what are we eating today* product combinations and provide recipes. |
| **Presentation & information** | **Signage on shelf-tags with symbols** | Introduction of signage theme via symbols, focussing on three main themes: Taste, convenience, and favourite product. |
|  | **Symbols on shopping carts handles, checkout bars and checkout screens** | Priming and reminders of theme with symbols (taste, convenience, and favourite product). |
|  | **Static stickers on glass cooling doors** | Improve product visibility through signage and symbols. |
|  | **Shelf tags** | Product promotion cards with vivid hedonic descriptions (e.g., “Dynamite beets”, “Twisted citrus-glazed carrots”) or indicating 'extra delicious' or 'extra refreshing', or with symbols indicating taste, short preparation time or sociability. Tag on the vegetables and fruits bags to promote a higher quantity (for example 'fill up to half of your bag with your favourite vegetables'). Promote product as a *favourite choice in this supermarket*. |
|  | **Shelf talker** | Improve product visibility through shelf banner with an image on it, defining for example "take it" or other terms, or seeing someone pictured already looking at/ grabbing a product. |
|  | **Food preparation tips/steps** | Increasing the attractiveness of self-cooking / vegetables by showing already prepared products, or with drops (freshness), moving food, with first person perspective, or to make in 5 min/ 3 steps, at the vegetable department. Explanation could be with recipe. |
| **Functionality** | **Dynamic nudge** | Sensor to detect that someone is standing for the shelf, on which shelf then reacts to encourage people to buy more (different) vegetables & fruit. |
|  | **Feedback on healthy choice** | Feedback on choice by making a message on the shelf; for example, ‘Good job!’ is visible when a product is grabbed out of the shelf. |
|  | **Placemat** | Placemat in bottom of cart or basket can indicate certain food proportions on a plate by means of stripes (at least half filled with vegetables), or can contain images of a simply prepared dish in which ingredients are easily recognizable. For example, 10 different versions can be created. |

**Table S3. Outline for implementation fidelity checklist**

|  | **In-store location of intervention** | **Score** | | |
| --- | --- | --- | --- | --- |
| ***Nudges*** |  |  | | |
| **End of aisle** | (all relevant end of aisle) | 0 | 0,5 | 1 |
| **In aisle baskets** | (all relevant baskets) | 0 | 0,5 | 1 |
| **Product shelf ratio and arrangement** | (all relevant shelfs) | 0 | 0,5 | 1 |
| **Check-out shelf** | (all relevant shelfs) | 0 | 0,5 | 1 |
| **Shelf-tags** | (all relevant shelfs) | 0 | 0,5 | 1 |
| **Shelf-talker** | (all relevant shelfs) | 0 | 0,5 | 1 |
| **Static stickers on glass cooling doors** | (all relevant shelfs) | 0 | 0,5 | 1 |
| **Feedback plank statics** | (all relevant shelfs) | 0 | 0,5 | 1 |
| **Symbols on:** | All shopping carts handles | 0 | 0,5 | 1 |
|  | All checkout bars | 0 | 0,5 | 1 |
|  | All check-out screens | 0 | 0,5 | 1 |
| **Food preparation tips/steps** | Posters at vegetables and fruits | 0 | 0,5 | 1 |
| **Symbols in shopping basket** | All baskets | 0 | 0,5 | 1 |
| **‘What are we eating today’ inspiration** | Suggests healthy swap | 0 | 0,5 | 1 |
| **(Dynamic nudge)** | Fruits and vegetable | 0 | 0,5 | 1 |
| ***Pricing strategies*** |  |  |  |  |
| **Price increases on price tags** | (all relevant shelfs) | 0 | 0,5 | 1 |
| **Price promotions on promotion-tags** | (all relevant shelfs) | 0 | 0,5 | 1 |
| Total score | |  | | |
